# Ionic Liquid Aqueous Two-Phase Systems From a Pharmaceutical Perspective

**DOI:** 10.3389/fchem.2019.00135

**Published:** 2019-03-15

**Authors:** Lisa McQueen, David Lai

**Affiliations:** ^1^Drug Product Design and Development, GlaxoSmithKline, Collegeville, PA, United States; ^2^Product and Process Engineering, GlaxoSmithKline, Collegeville, PA, United States; ^3^Advanced Manufacturing Technologies, GlaxoSmithKline, Collegeville, PA, United States

**Keywords:** aqueous two-phase systems, biphasic systems, ionic liquids, pharmaceutical extractions, pharmaceutical separations

## Abstract

Aqueous Two-Phase Systems (ATPSs) have been extensively studied for their ability to simultaneously separate and purify active pharmaceutical ingredients (APIs) and key intermediates with high yields and high purity. Depending on the ATPS composition, it can be adapted for the separation and purification of cells, nucleic acids, proteins, antibodies, and small molecules. This method has been shown to be scalable, allowing it to be used in the milliliter scale for early drug development to thousands of liters in manufacture for commercial supply. The benefits of ATPS in pharmaceutical separations is increasingly being recognized and investigated by larger pharmaceutical companies. ATPSs use identical instrumentation and similar methodology, therefore a change from traditional methods has a theoretical low barrier of adoption. The cost of typical components used to form an ATPS at large scale, particularly that of polymer-polymer systems, is the primary challenge to widespread use across industry. However, there are a few polymer-salt examples where the increase in yield at commercial scale justifies the cost of using ATPSs for macromolecule purification. More recently, Ionic Liquids (ILs) have been used for ATPS separations that is more sustainable as a solvent, and more economical than polymers often used in ATPSs for small molecule applications. Such IL-ATPSs still retain much of the attractive characteristics such as customizable chemical and physical properties, stability, safety, and most importantly, can provide higher yield separations of organic compounds, and efficient solvent recycling to lower financial and environmental costs of large scale manufacturing.

## Introduction

The adoption of methods from the scientific literature into industrial applications often follows a period of dormancy. While ATPSs have experienced a recent prolific rise in applications in microfluidics, cellular engineering, bioprinting, and biopatterning since the 2000s (Teixeira et al., 2017), the industrial applications of ATPSs are typically separations and purification, first described in the 1950s (Albertsson, 1956, 1958). Such ATPSs popularized by Albertsson are polymer-polymer or polymer-salt emulsions that have been well-studied for viral (Norrby and Albertsson, 1960; Liu et al., 1998; Effio et al., 2015), cellular (Walter et al., 1976; Sharp et al., 1986; Kumar et al., 2001), nucleic acid (Ribeiro et al., 2002; Gomes et al., 2009; Nazer et al., 2017), protein (Schmidt et al., 1994; Balasubramaniam et al., 2003), and antibody (Desbuquois and Aurbach, 1971; Selber et al., 2004; Rosa et al., 2007b; Azevedo et al., 2009a) separations. Examples can be found for batch (Tavana et al., 2009; Frampton et al., 2011, 2014; Lai et al., 2011) or continuous (Yamada et al., 2004; Nam et al., 2005; SooHoo and Walker, 2009; Tsukamoto et al., 2009; Rosa et al., 2012) processes. While polymer ATPSs are limited to partitioning of macromolecules due to size of the polymers used, the isolation and separation of small molecules (< 900 Daltons) in ATPSs, especially those developed in the pharmaceutical industry, commonly employ ionic liquids (ILs).

### Polymer-Based ATPS Separations and Considerations

**Figure 1:**
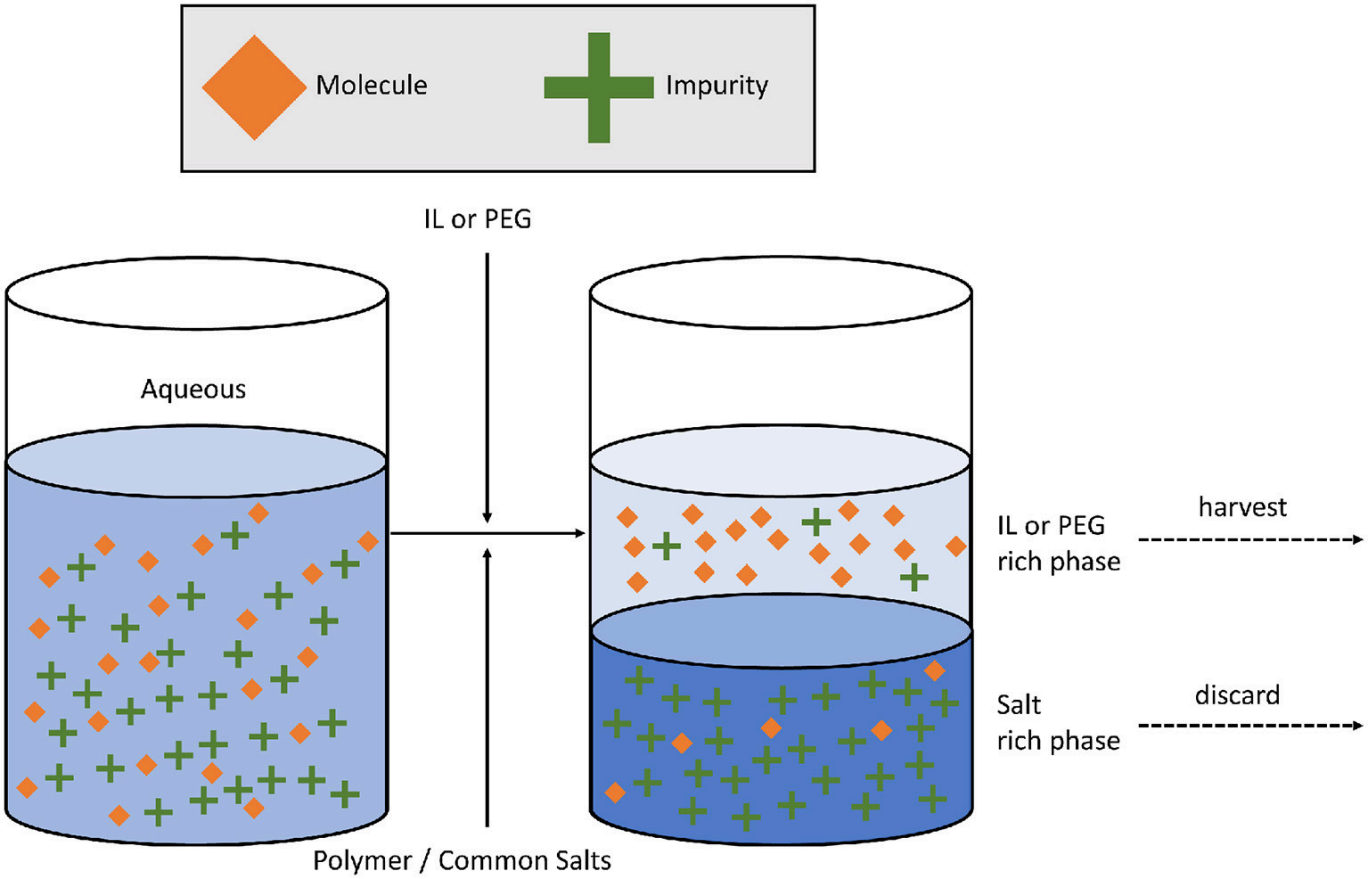
The use of polymer-based or IL-based ATPSs for separations critically depends on selective partitioning of the molecule over impurities. Impeller agitation is often employed to speed up equilibrium partitioning. Depending on viscosity and density differences and interfacial tension values, physical separation can be as simple as sedimentation or as complex as industrial centrifugation to isolate the liquid phase containing the molecule of interest.

The mechanism for purification of a material using ATPSs is driven by physical and chemical affinity toward a select phase and the contaminant's affinity toward the other liquid phase. Accurate prediction of partitioning is complicated by several factors. Physical and chemical properties of each phase, such as viscosity, relative volume, density, charge, pH, and volatility are known to impact performance, and thus the choice of polymer or salt is used to tune the systems for effectiveness (Albertsson, 1960). In general, the process of ATPS separations and purification occurs in three major steps: molecular partitioning, physical separation, and isolation of phase of interest (Figure 1). The use of ATPSs was shown to be a high-yield and environmentally sustainable alternative to some current pharmaceutical purification and extraction processes (Chen et al., 2005). Although its adoption in the pharmaceutical industry is relatively early for manufacturing, it currently has its niche applications in pharmaceutical development as well as other industries (Diuzheva et al., 2018; Mocan et al., 2018). Clear documentation of advantages in cost savings, yield and sustainability combined with ease of adoption is needed to ease its widespread use in biopharmaceutical or vaccine manufacturing process (Chen et al., 2005).

Selective partitioning is the first step of separation design. It is advantageous to increase the liquid-liquid interface to accelerate molecular partitioning by producing a fine emulsion of the dispersed aqueous phase inside the continuous aqueous phase. The ability to selectively partition is dependent on the affinity differences for the material of interest for each phase. Polyethylene glycol (PEG) is a widely used component of ATPSs due to its high biocompatibility, biodegradability, water solubility, and low cost. Aside from changes in molecular weight, the properties of PEG with respect to partitioning are limited. To overcome this challenge, multiple groups have functionalized PEG in PEG/salt and PEG/dextran ATPSs with glutaric acid to improve extraction yields of immunoglobins from 28 to 93% (Rosa et al., 2007a), and 23 to 97% (Azevedo et al., 2009b) as well as increasing extraction efficiencies of penicillin up to 96% using imidazole-terminal PEG (Jiang et al., 2009). The addition of a small percentage of a biospecific ligand to ATPSs was shown to increase purification efficiency and yield by several fold (Kula et al., 1991) in a concentration-dependent manner that can be optimized for manufacturing scale of monoclonal antibodies. The use of ATPSs present an opportunity to simplify the manufacturing process of plasmid DNA by allowing for the lysis, recovery, purification and extraction in a single high yield step (Frerix et al., 2005). Polymer ATPSs have been shown to extract plasmid DNA and RNA up to 90 and 70% respectively (Frerix et al., 2006).

The second step is physical coalescence of the emulsion into two continuous, phase-separated, liquids. While numerous physical and chemical properties drive selective partitioning, coalescence of ATPS is simply driven by interfacial tension, density and viscosity. ATPSs will spontaneously coalesce into two continuous phases over time by sedimentation according to Stoke's Laws, and physical characteristics such as viscosity slow the process (Asenjo and Andrews, 2012). Centrifugation is often used to accelerate the natural coalescence and sedimentation.

Lastly, the liquid phase containing the purified material is isolated. The interface between the two liquid phases is avoided to prevent contamination, thus resulting in a yield loss. Multi-stage ATPS separations can improve recovery (Benavides et al., 2006) and may be justifiable depending on the relative ATPS cost and compound value. Vessels with a high aspect ratio are recommended to minimize the liquid-liquid interface where compound loss occurs, however homogenization in a high aspect ratio vessel generally has longer mixing times or suffers from undesirable fluid compartmentalization (Magelli et al., 2013). Furthermore, tall vessels result in long centrifugal distances and longer sedimentation times to achieve complete separation.

### Polymer-Based ATPS Pharmaceutical Industry Applications

After fermentation, separation of API from cells and cell debris is often achieved *via* centrifugation, however centrifugation becomes increasingly complex on scale up from bench (50 mL) to industrial scale (1,000 L) (Majekodunmi, 2015). Thus, there exists a need to explore advanced scalable separation processes that maintain yield for a single-step extraction. Polymer-based ATPSs are showing promise for a wide range of commercially valuable molecules, however their versatility also complicates its adoption. The principles governing high-value molecule partitioning has been investigated (Willauer et al., 2002) and the consultation of such toolboxes (Huddleston et al., 1999) to assist in ATPS design and development is highly encouraged.

One other factor that prohibits the attractiveness of polymer-polymer ATPSs is the cost of high molecular weight polymers at industrial scale (Torres-Acosta et al., 2018). As a result, cheaper phosphate or sulfate salts as replacements for polymer-polymer ATPSs to become polymer-salt ATPSs are more attractive. Such polymer-salt ATPSs were demonstrated to have high yield and scalability from bench to industrial scale (Hart et al., 1994). The substitution of a high molecular weight polymer with a salt, introduces an osmotic pressure concern which limits ATPS application in some cellular and biomolecular recoveries (Kühn, 1980; Zijlstra et al., 1996; Li et al., 2000). Despite this, in certain products with challenging yields, ATPSs have financial justification at the industrial scale especially with the reduced costs of polymer-salt ATPSs and IL-ATPSs (Torres-Acosta et al., 2018).

## Ionic Liquid ATPS

Ionic liquids have similar tunable properties such as hydrophobicity, polarity, solvation, and phase separation (Greaves et al., 2006; Moreno et al., 2015; Anderson and Clark, 2018). The stark similarities of polymer-based and IL-based ATPSs have facilitated the adoption of IL-based ATPSs. IL-ATPSs provide improvements in separation performance, selectivity, and broader design opportunities for a diverse range of molecules, including small molecules and biomolecules. IL-ATPSs also possess lower molecular weight and viscosity which enables faster separations making them industrially attractive. We note that the separation of cells, which is frequently performed in drug discovery experiments, and an obvious area for improvement through use of IL-ATPSs is not possible or demonstrated with IL-ATPSs, to the best of our awareness, likely due to osmotic pressure effects.

While this review provides a pharmaceutical focus, the reader is encouraged to reference other IL-ATPS reviews that informatively summarize properties for partitioning (Li et al., 2010; Oppermann et al., 2011; Ma et al., 2018), phase behavior and IL recovery (Freire et al., 2012), enzyme separations (Nadar et al., 2017), deep eutectic solvents (DES) (Shishov et al., 2017; Zainal-Abidin et al., 2017), and capillary electrophoresis applications (El-Hady et al., 2016). A review of various roles of ILs in pharmaceutical applications is also recommended (Huddleston et al., 1998).

Except where noted, all processes, physical properties and performance information described were generated at 25°C.

## Types and Properties of IL-ATPS

Phase separation can be controlled by the differing nature of the IL and second phase hydrogen bonding properties (Bridges et al., 2007). The IL-rich environment, often the upper phase, is relatively more hydrophobic thus attracting hydrophobic molecules. Phase separation is thus understood to be solvophobic and driven by electrostatic attractions and hydrophobic interactions. Several different systems have been discovered, characterized, and investigated for their potential applications in the pharmaceutical industry. IL-ATPSs can be tuned to improve solubility and thus extraction efficiency and partitioning properties which in turn improves speed and completion of phase separation. Generally, the lower the salt concentration required, the stronger the phase separation ability of the ILs from aqueous solutions of salts.

### IL Cation

C_n_-methyl-imidazolium based ILs, where *n* = 2, 4, 6, and 8, are the most frequent systems studied in the literature, along with other functionalized imidazolium cores (Gutowski et al., 2003; Wu et al., 2015). Other systems such as cholinium based carboxylate ILs (oxalate, malonate, succinate, L-malate, fumarate, glutarate, and citrate), and pyridinium based -ILs are described with PEG 400 and 600 as the second phase former (Passos et al., 2012; Mourão et al., 2014). Varying CH_2_ length resulted in the coexistence of distinct types of self-assembly in the IL-ATP system. The behavior was inconsistent on changing the IL anion. For example, increasing the imidazolium alkyl chain length increased phase separation for a series of BF${}_{4}^{-}$ ILs and decreased for the same series as Br^−^ ILs (Wei et al., 2013). IL-ATPS and their applications were also demonstrated for dodecyl trimethylammonium-based (Flieger et al., 2013), functionalized amino acid and nucleotide ILs, for example glycine and guanidine (Wu et al., 2013; Zeng et al., 2013; Ding et al., 2014; Yao et al., 2016). Phosphonium-based ILs have the advantage of being less dense than water and less viscous than imidazolium based ILs, thus prospectively facilitating the separation of the aqueous waste stream of biomolecule separations in production scale equipment (Louros et al., 2010).

### IL Anion

Several different IL counterions have been described in the IL-ATPS literature, although simple anions such as bromide and chloride are most typical. Amino acid anions, L -serine, L-glycine L-alanine, and L-leucine with [bmim] cations formed IL-ATPS with K_3_PO_4_ (Wu C. et al., 2011). The ability to form IL-ATPS was related to hydrophobicity of the amino acid anion such that phase formation occurs with increasing hydrophobicity of the anion. Formate, acetate, propionate, lactate, chloride, and bromide salts of functionalized guanidinium ILs were shown to be effective at protein partitioning (Ding et al., 2014). Antioxidant anions (butanoate, propanoate, acetate, lactate, glycolate, bitartrate, dihydrogen phosphate, chloride, dihydrogen citrate, gallate, syringate, vanillate, and caffeate) of cholinium ILs were effective in separating immunoglobulin G (Ramalho et al., 2018).

### ATPS-Forming Salts

IL-ATPSs can be formed with conventional kosmotropic salts (potassium sulfate, citrate, ammonium sulfate), common salts (citric, fumaric, succinic, and tartaric acid sodium salts), amino acids, PEG and surfactants (3-p-nonylphenoxy-2-hydroxypropy trimethyl ammonium bromide, NPTAB and sodium dodecyl sulfonate, SDS), and combinations (Wu C. et al., 2011; Passos et al., 2012; Wei et al., 2013; Abdolrahimi et al., 2015; Dai et al., 2015; Pereira et al., 2015). The size of the biphasic region can be controlled by functionalizing the PEG terminal groups with –OH, –OMe, or –NH_2_ to change the hydrogen bonding donor or acceptor. Improvements in the extraction performance for a hydrophobic molecule, tryptophan was demonstrated in citrate-based IL-ATPSs for use in continuous extraction of amino acids. The incorporation of surfactants decreased the viscosities of both phases and flipped the IL-rich phase to the bottom phase as a result of density changes (Wei et al., 2013; Dai et al., 2015).

The molecular mechanisms that govern the ability of salt ions to induce IL-ATPS formation was studied using a wide range of salts with diverse combinations of cations and anions, with [bmim][CF_3_SO_3_] a hydrophilic ionic liquid (Shahriari et al., 2012). The ions' trend to salt-out the IL into an ATPS follows the Hofmeister series and demonstrates the molar entropy of salt ion hydration as the driving force.

### Additives

Addition of a third component to create an IL-ATPS is another tactic used to enhance separation, presumably by increasing the solvophobic nature of the systems. For example, addition of SDS to [bmim]Br/NaCl IL-ATPS enhanced antibiotic extractions into the surfactant-rich upper phase (Yang et al., 2014). Addition of CO_2_ into aqueous solutions of ILs and amines was shown to be effective in recovering ILs from aqueous mixtures (Xiong et al., 2012). Simply by bubbling CO_2_ at atmospheric pressure into aqueous IL forms an IL-ATPS and allowed up to 99% recovery of the IL. Interestingly, these systems also modified densities such that the IL-rich phase is at the bottom. They also found phase separation of ammonium salts and the recovery efficiency for ILs are predominantly driven by the pKa of the amine, following the order: 1,2-propylenediamine > monoethanolamine > diethanolamine > N-methylmonoethanolamine > N-ethyldiethanolamine > triethanolamine, except for > N-methyldiethanolamine.

Application of an electrokinetically stable three-phase IL-ATPS, [bmim]Cl/ K_2_HPO_4_, [bmim]BF_4_/ NaH_2_PO_4_, and [bmim]BF_4/_ Na_3_(citrate) enabled electrokinetic de-mixing (Li et al., 2017). Increased salt concentration and decreased IL concentration were observed to phase separate faster and have a higher efficiency of electrokinetic demixing due to a salting-out effect, whereas electric field had no impact on protein distribution.

In addition, pH-dependent reversible partitioning of IL-ATPS systems at ambient conditions offers dual functionality as a catalytic medium and separation extraction (Ferreira et al., 2017). Adjustment of pH was achieved through changes in salt speciation or bubbling in CO_2_ or NH_3_.

## IL Extractions

### Small Molecule Separations

Several types of small molecules such as antioxidants, flavonoids, alkaloids, sulfonamides, functionalized amino acids, antibiotics such as tetracycline, oxytetracycline, cephalexin, and chloramphenicol effectively partition and extract in IL-ATPSs (Li et al., 2005; Freire et al., 2010; Louros et al., 2010; Han et al., 2011; Berton et al., 2012; Lin et al., 2012; Wu et al., 2013; Mourão et al., 2014; Yang et al., 2014; Abdolrahimi et al., 2015; Yao and Yao, 2017). In general, small molecules partition into the IL-rich upper phase. These systems are advantageous as a sample pre-treatment technique offering reduction of steps, materials and volatiles, and a sample concentration method (Basheer et al., 2008). IL-ATPSs have broad application in selective separation and extraction of small molecules and biomolecules through simple modifications of the IL structure and composition along with parameters that affect the extraction efficiency, such as the salt concentration, pH and extraction temperature, to appropriately modulate the IL-rich phase.

Pei et al. determined the partition coefficients for a series of amino acids in [C_n_mim]Br (*n* = 4, 6, 8)/ K_2_HPO_4_ IL-ATPS and showed that they increased with increasing hydrophobicity of the amino acids and ionic liquids, solution pH value, tie-line length of the ATPSs and temperature (Pei et al., 2012). Pre-treatment of aqueous solutions for trace analysis of chloramphenicol with a 147-fold concentration enrichment and high recovery was achieved by applying an electric field across the IL-ATPS (Yao and Yao, 2017). A simple and sensitive method for selective extraction of quinine from human plasma was developed with [bmim]Cl/potassium phosphate buffer (Flieger and Czajkowska-Zelazko, 2015).

Extraction of pharmaceutical molecules (active ingredients or intermediates) is often from plant materials. The isolation of high purity secoisolariciresinol diglucoside, a multi-functional pharmaceutical, from flaxseed, was simplified and improved with an ultrasonic-assisted extraction followed by IL-ATPS formation by the addition of Na_2_SO_4_ (Tan et al., 2015). A similar example where fractionation and recovery of bioactive hydroxycinnamic derivatives from stressed carrot mass using IL-ATPS formed from [emim]acetate/potassium buffers gave a different phenolic ratio than conventional ATPS (Sánchez-Rangel et al., 2016).

Enantiomeric separation of 7 racemic amino acid mixtures was achieved by designing task-specific hydrophilic hexafluorophophate IL-ATPSs (Wu et al., 2015). Enantioselectivity was dependent on IL structure with energetically-favored intermolecular interactions between the IL cation, water and the D-enantiomer, allowing separation of the D-enantiomer to the bottom IL-rich phase. The IL-ATPS extraction efficiency was further improved for D,L-phenylalanine using chiral tropine-based ILs, [C_n_Tropine]proline (*n* = 2, 3, 4, 5, 6, 7, 8) with Cu(acetate)_2_ and potassium phosphate buffers (Wu et al., 2015).

### Macromolecule Extractions/Analytical Biochemistry

The introduction of IL-ATPSs is a significant advancement in analytical biochemistry in terms of time, materials, complexity and efficiency for purification and separation of macromolecules in aqueous fluids. Due to the high water content in both phases, they are biocompatible media for the extraction and purification of a wide range of biomolecules by offering protection from denaturation. Separation is attributed to electrostatic potential differences between the coexisting phases, hydrophobic, and hydrogen bonding interactions of the biomolecules with the IL components in the upper IL-rich phase, and the salting-out effect.

Proteins, such as hemoglobin, ovalbumin, bovine serum albumin (BSA), bovine hemoglobin proteins, lysozyme, cytochrome c, and trypsin appear to require task-specific ILs per entity for successful separation and efficiencies as high as 99.6% (Lin et al., 2013; Wu et al., 2013; Zeng et al., 2013; Ding et al., 2014; Wang et al., 2015; Xu et al., 2015; ČíŽová et al., 2017). Extraction efficiencies are influenced by the amount of IL, the concentration of salt solution, temperature, and protein concentration. Separations were driven by hydrophobic interactions between IL cations and the proteins with minor contributions from electrostatic interactions and the salting-out effect.

In addition to separation, IL-ATPS were also used to improve binding constant estimation by stabilizing the protein of interest (El-Hady et al., 2015; Abd El-Hady and Albishri, 2018). For example, binding constants of (+)-propranolol, methotrexate and vinblastine to human alpha (a1)-acid glycoprotein, the plasma protein were determined by combining affinity capillary electrophoresis with reversible temperature-dependent phase separation using [bmim]Cl/ phosphate IL-ATPS with improved precision.

The antibody, immunoglobulin G (IgG) was extracted from serum samples using cholinium based IL-ATPSs (Ramalho et al., 2018). Although some systems had superior IgG recovery values to conventional techniques, improvement in IgG purity levels was insignificant. IL-ATPS represents a far simpler separation method when compared to current multistep methods for antibody isolation from serum samples. Such technology is worth further development and optimization.

## Catalytic Enhancement

IL-ATPS also found utility as catalyst performance enhancers that offer a dual advantage in synthetic processes when selective separation is considered (Dyson et al., 2001; Wu D. X.et al., 2011; Mai and Koo, 2014). The bioconversion of the steroid 16a,17-epoxyprogesterone to 11a-hydroxy-16a, 17-epoxyprogesterone catalyzed by free *R. nigricans* cells was improved when performed in an IL-ATPS without the addition of salt, and demonstrated in semi-continuous production for 60 h at ~5 mL (total) scale (Wu D. X.et al., 2011). A temperature-dependent reversible IL-ATPS was demonstrated as a medium for transition metal catalyzed hydrogenation of a water-soluble substrate, offering potential re-use of the high-value catalyst and avoidance of product metal contamination (Dyson et al., 2001). Similarly, temperature-controlled reversible phase separation offers the advantage of product recovery and maintaining catalytic activity of an *in-situ* penicillin G hydrolysis (Mai and Koo, 2014). In keeping with the well-known trends of enzymatic activity in ILs, catalytic activity was found to increase in IL hydrophobicity.

## Conventional and IL-ATPS Crossover

**Figure 2:**
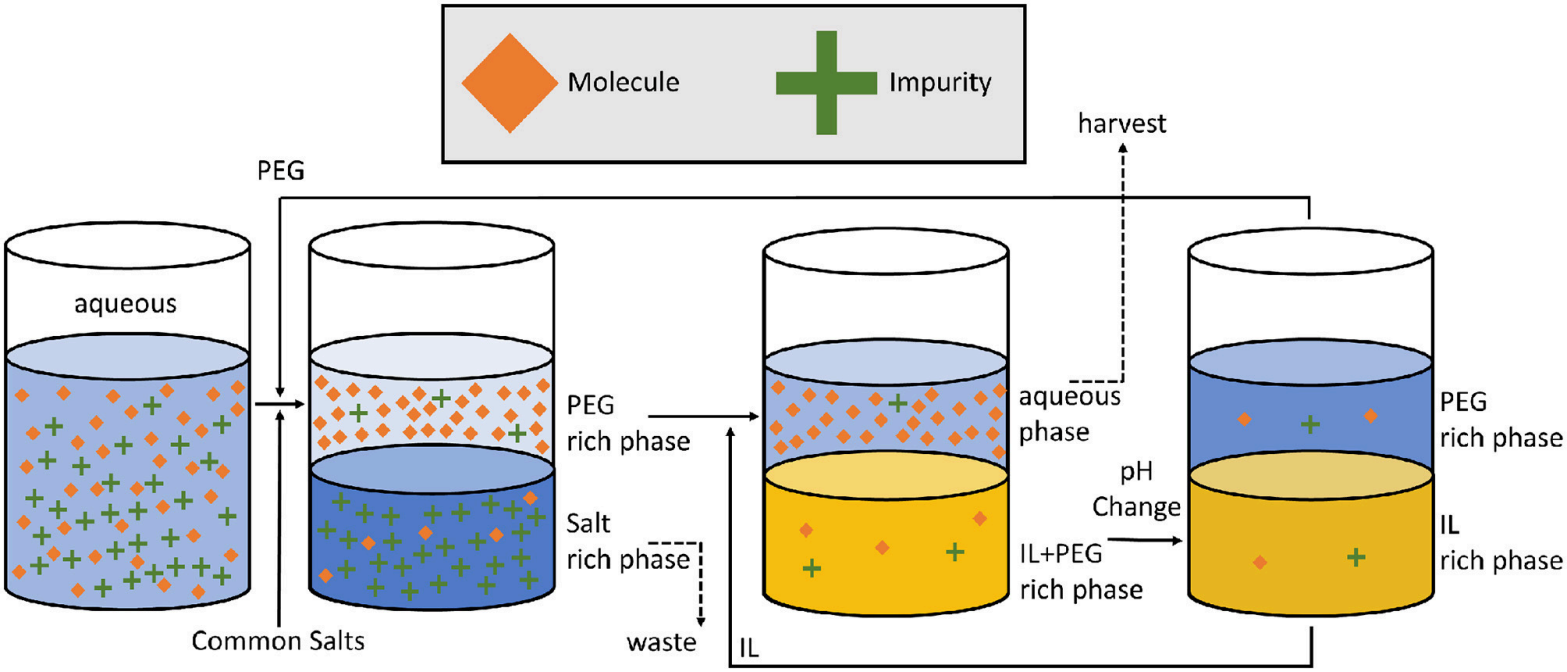
IL assisted polymer recovery for ATPS extraction proposed by Jiang et al. (2009). The recent merge between IL and conventional ATPSs creates new opportunities to further decrease the carbon footprint of ATPS extractions which also result in cost savings. Such financial and environment drivers are key to industrial adoption.

There are a few examples of note where polymer-based ATPS were combined with ILs. Extraction efficiency of three antioxidants, gallic, vanillic, and syringic acids, was further improved by tuning the polarity of the PEG-rich bottom phase with the addition of an IL (Almeida et al., 2014). The relationship between hydrogen bonding of the IL with the phenolic acids and extraction was found to be more important than the total amount of IL and increasing the polymer molecular weight increased the likelihood of phase separation. Tropine-salt IL-ATPSs were used to remove or recover hydrophilic ILs that cannot form IL-ATPSs with kosmotropic salts, polymers or carbohydrates, in an attempt to provide a means to reduce environmental contamination (Wu et al., 2016). The ILs preferentially partitioned to the tropine-rich upper phase, which is then further processed to complete the recovery. IL-ATPSs have also been employed for polymer recovery and recycling (Jiang et al., 2009) which offers the benefits of cost and carbon footprint reduction and may be key to wider industrial adoption (Figure 2).

## Forward Statement

ATPSs have already found industrial use in niche areas of pharmaceutical development and possibly manufacturing. Meanwhile, ILs are simultaneously emerging as sustainable alternatives for small molecule pharmaceutical extractions. The recent development of IL-based ATPSs provides new opportunities for decreasing the carbon footprint and costs of pharmaceutical extractions which are important drivers for wider industrial adoption. The body of literature is clear that IL-based and polymer-based ATPSs are suitable sustainable alternatives to organic solvents and produce higher or equivalent yields. We believe the barriers to industrial adoption have been cracked due, in part, to the scientific works highlighted in this review, and that there is a bright pharmaceutical future for polymer and IL-based ATPSs.

## Author Contributions

LM provided review on ILs and DL provided review on polymer-based ATPS processes.

### Conflict of Interest Statement

LM and DL are employed by GlaxoSmithKline.
